# Deep Convolutional Neural Network Phase Unwrapping for Fringe Projection 3D Imaging

**DOI:** 10.3390/s20133691

**Published:** 2020-07-01

**Authors:** Jian Liang, Junchao Zhang, Jianbo Shao, Bofan Song, Baoli Yao, Rongguang Liang

**Affiliations:** 1 State Key Laboratory of Transient Optics and Photonics, Xi’an Institute of Optics and Precision Mechanics, Chinese Academy of Sciences, Xi’an 710119, China; liangjian@opt.ac.cn (J.L.); yaobl@opt.ac.cn (B.Y.); 2 James C. Wyant College of Optical Sciences, University of Arizona, Tucson, AZ 85721, USA; junchaozhang@email.arizona.edu (J.Z.); jianboshao@email.arizona.edu (J.S.); songb@email.arizona.edu (B.S.)

**Keywords:** phase unwrapping, deep learning, 3D imaging

## Abstract

Phase unwrapping is a very important step in fringe projection 3D imaging. In this paper, we propose a new neural network for accurate phase unwrapping to address the special needs in fringe projection 3D imaging. Instead of labeling the wrapped phase with integers directly, a two-step training process with the same network configuration is proposed. In the first step, the network (network I) is trained to label only four key features in the wrapped phase. In the second step, another network with same configuration (network II) is trained to label the wrapped phase segments. The advantages are that the dimension of the wrapped phase can be much larger from that of the training data, and the phase with serious Gaussian noise can be correctly unwrapped. We demonstrate the performance and key features of the neural network trained with the simulation data for the experimental data.

## 1. Introduction

**Figure 1:**
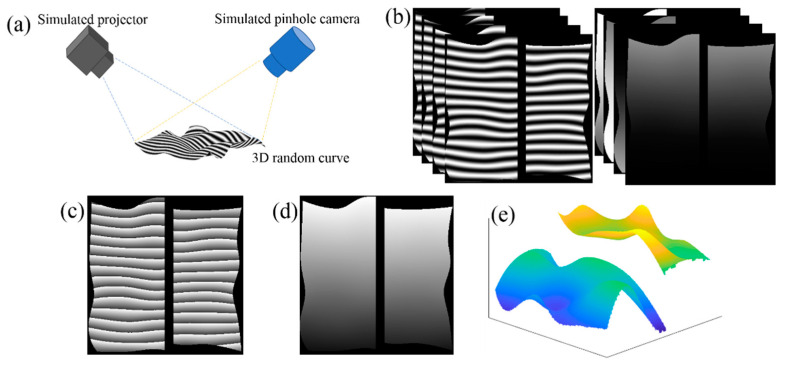
Fringe projection 3D imaging. (**a**) Typical system configuration, (**b**) two sets of isolated surface images modulated by phase-shifted fringes with different pitches, (**c**) wrapped phases, (**d**) unwrapped phases, and (**e**) final unwrapped phase.

Fringe projection 3D imaging is a widely used technique for shape measurement [1,2,3,4,5]. As shown in Figure 1, by projecting three or more phase-shifted structured light, typically linear sinusoidal patterns, onto the surface under the measurement, multiple images of the surface with distorted phase-shifted fringes can be captured to estimate the surface shape. The phase calculated from the images modulated with phase-shifted fringes is wrapped to the range of (−*π*, +*π*] due to the inverse trigonometric computation. One essential step in 3D imaging with fringe projection method is to unwrap the wrapped phase. Various phase unwrapping approaches have been developed in past few decades to improve the speed and accuracy [6,7,8,9,10,11,12].

Recently deep learning methods have been proposed as an alternative approach for phase unwrapping to improve the speed and accuracy [13,14,15,16,17,18]. Some of the proposed methods are based on the use of a residual neural network [13,17] to directly perform the unwrapping task. These methods can obtain the unwrapped phase directly, but with relatively large errors. Others are based on the segmentation neural network [14,15], where the strategy is to segment the wrapped phase and label each segment with an integer, and then train the neural network to label each segment. While those deep learning based phase unwrapping methods have different degrees of success in their targeted fields, no general deep learning based method is available to address the phase unwrapping problems in phase-shift fringe projection 3D imaging.

Due to the special properties in the wrapped phase, as shown in Figure 1, the reported deep learning phase unwrapping methods are unsuccessful in unwrapping phase in fringe projection 3D imaging. There are two major differences in the wrapped phase map of the fringe projection compared to those of interferometry [15]. As shown in Figure 1a, the linear fringes are projected onto the surface from one direction relative to the camera, the calculated phase is typically wrapped in one-direction, depending on the relative position of the camera and the projector. While it is easy to use the segmentation network to segment the wrapped phase, it is difficult to label each segment due to the similarity between the segments as shown in Figure 1c. Another key feature in fringe projection is that typically two sets of measurement are needed for high resolution and large depth range 3D imaging: one set of the measurement with wide fringe pitch is used to solve the discontinuity issue and the other set of the measurement with narrow fringe pitch to achieve high resolution depth imaging. Therefore, a solution is needed to combine the measurement results from the two measurements to obtain the accurate measurement.

In this paper, a new phase unwrapping segmentation network is introduced for fringe projection 3D imaging. The wrapped phase will be segmented first and then each segmented phase is labeled with four key transition points as the training data for training the first neural network (network I). Then, the outputs from the first neural network will be labeled as the inputs to train the second neural network (network II). Finally, a simple algorithm will be used to obtain the unwrapped phase from the output of the trained neural network. The effectiveness of the proposed phase unwrapping method has been demonstrated by the simulation and experimental data.

## 2. Proposed Method

The unwrapped phase is the sum of the wrapped phase and the appropriate integer multiple of 2*π* in each wrapped region, which can be expressed as:

(1) $$\phi_{U}\left(x,y\right)=\phi_{W}\left(x,y\right)+2\pi \cdot k\left(x,y\right),$$

where *ϕ_W_* and *ϕ_U_* are the wrapped and unwrapped phase, respectively, *k* indicates the integer multiple of 2*π*, (*x*, *y*) is the spatial coordinates of the pixel. In [14,15], *k*(*x*, *y*) is set as the training output instead of *ϕ_U_*(*x*, *y*), which converts a regression problem into a semantic segmentation problem. This approach is not suitable for unwrapping phase in 3D fringe projection imaging because the segmented phases have similar feature as shown in Figure 1c, the trained neural network cannot label each segmented phase an appropriate integer [14,15]. In addition, the wrapped phase is often discontinuous as shown in Figure 1c, where two isolated objects are at different locations.

**Figure 2:**
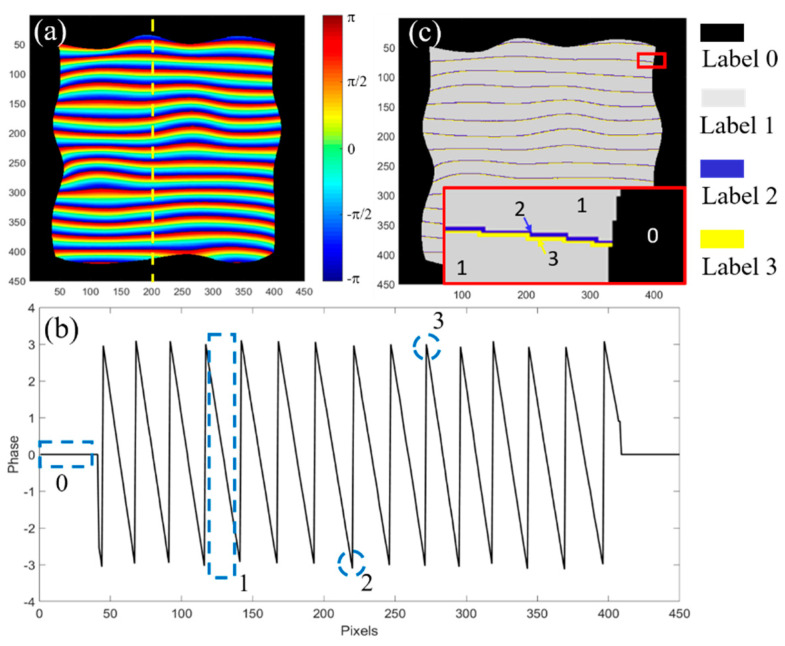
Four features of the wrapped phase for unwrapping phase. (**a**) wrapped phase, (**b**) phase distribution of one column indicated by the yellow dashed line in (**a**), and (**c**) segmented wrapped phase with labels.

To develop an effective phase unwrapping neural network, it is necessary to consider the specific properties of the wrapped phase in fringe projection. A wrapped phase of a random 3D surface calculated by four-step phase shifting method is shown in Figure 2a. Each wrapped period looks similar, meaning there are little features to be distinguished from other periods. Figure 2b plots the phase distribution of one column indicated by the yellow dash line in Figure 2a. There are four features which can be used for unwrapping phase: background (Label 0), segmented phase region (Label 1), bottom transition point (Label 2) and peak transition point (Label 3). Figure 2c plots the segmented wrapped phase labeled with four features. To intuitively illustrate the transition points, the inset in Figure 2c shows one period of the wrapped phase with the labels in the corresponding position.

**Figure 3:**
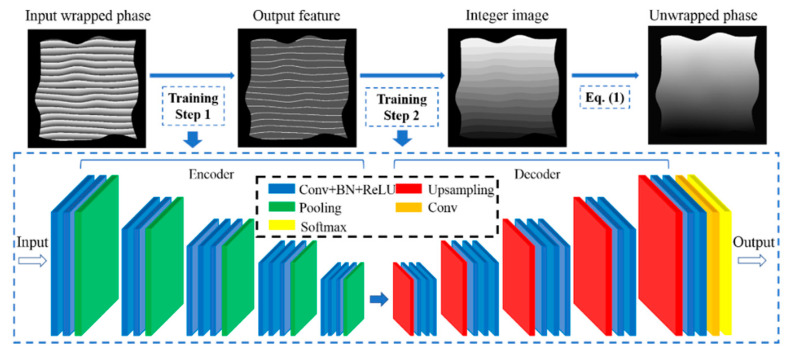
The workflow of the proposed method and the network architecture for phase unwrapping.

Figure 3 shows the workflow and the architecture of the proposed convolutional neural network. The network contains an encoder path, a decoder path and a pixel-wise classification layer. The encoder path has five layers, and each layer has operations of convolution (Conv), batch normalization (BN), rectified linear unit (Relu) and max-pooling for down-sampling. Likewise, the decoder path also has five layers corresponding to the encoder path. The difference in the decoder path is the up-sampling operation. The up-sample maps are realized by using the corresponding memorized max-pooling indices in the encoder path. Note that, both the encoder path and the decoder path have 13 convolutional layers, which is exactly the first 13 layers in the VGG16 classification network [19]. The data output from the decoder path is then fed into another convolutional layer and finally output is obtained after a soft-max classifier. In the proposed network, 3-by-3 kernel size are chosen for all convolutional layers, 2-by-2 window and stride 2 for max-pooling operation.

In the training process, to measure the difference between the output and the ground truth (GT), cross entropy loss function is applied:

(2) $$loss=-\frac{1}{M}\sum_{k=1}^{M}\sum_{x,y}\log\left(p_{k,t}\left(x,y\right)\right),$$

where *M* is the number of the training dataset, *t* is the output class, *p_k_*_,*t*_ is the predicted probability. Adam optimizer is used with the batch size of 8. The learning rate is set to 0.0001, and the exponentially decays with a rate of 0.99. These parameters are chosen based on our previous work and detailed analyzed in [15].

As shown in Figure 3, the same network architecture is actually used twice with different outputs. Network I is trained to do the segmentation process, and the output is the 4-label segmented image; while network II is trained to assign the segmented image to the integer label. Finally, the unwrapped phase can be obtained using Equation (1). One major feature of the proposed deep learning based phase unwrapping method is that, in network I only 4 labels are used to characterize the wrapped phase information, the number of wrapped phase regions is unlimited. In addition, the size of the image is unlimited as well.

To train the network, the fringe projection and imaging process are simulated using the principle of the pinhole imaging, as shown in Figure 1. A classical four-step classic phase shift method is used to record four surface images modulated with phase-shifted fringes: *I*(*x*, *y*; 0), *I*(*x*, *y*; *π*/2), *I*(*x*, *y*; *π*) and *I*(*x*, *y*; 3*π*/2), where (*x*, *y*) is the coordinate of the pixels, as shown in Figure 1b. The inverse pinhole model is used to simulate a projector to project the stripe map onto an arbitrary surface, and the pinhole model to simulate a camera to trace the ray and form the image in the simulation. Thus, the phase *ϕ* can be calculated by these images, as:

(3) $$\phi \left(x,y\right)=\tan^{-1}\left(\frac{I\left(x,y;\;3\pi /2\right)-I\left(x,y;\;\pi /2\right)}{I\left(x,y;\;0\right)-I\left(x,y;\;\pi \right)}\right).$$

The phases of the 800 random surfaces are generated as the training dataset. The size of each phase image is 450 × 450 × 1, and the maximum number of wrapped phase regions is 17. 50 images from the training dataset are selected as the validation data, while another new 50 images are generated as the test data. The GT in Network I is generated from the wrapped phase directly according to the 4 features, while the GT in Network II is generated by manually filling integer to each segment.

**Figure 4:**
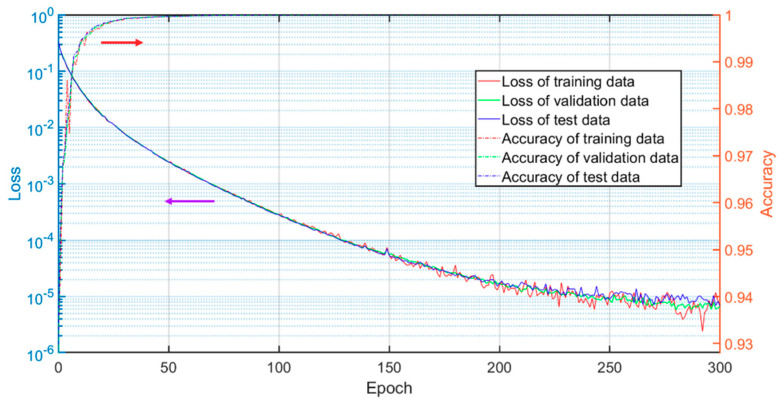
Loss and accuracy versus epoch during the training process.

The losses of the training dataset, the validation dataset and the test dataset are plotted in Figure 4. Note that Figure 4 shows the losses of Network II since the loss of the final output shows the accuracy of the entire process. As the epoch increases, the losses are reduced accordingly, and then converge after about 300 epochs, illustrating explicitly the effectiveness of the training process. As expected, the loss of the training data (red solid line) is the lowest of the three, while the loss of the test data (blue solid line) is the highest. The accuracy is calculated based on the root mean square error between the output and the GT. From Figure 4, it can be seen that the accuracies are very high after about 50 epochs, implying the proposed network architecture can effectively retrieve the features of the wrapped phase. The accuracy of the test data around 300 epochs is as high as 99.98%.

**Figure 5:**
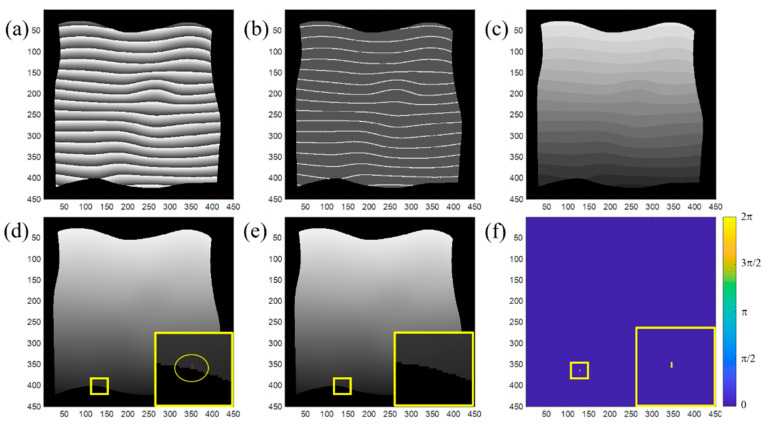
Phase unwrapping and 3D reconstruction. (**a**) Input data, (**b**) output phase feature map from network I, (**c**) phase feature map with assigned integer to each segment from network II, (**d**) unwrapped phase using the proposed method, (**e**) unwrapped phase using floodfill phase unwrapping method, and (**f**) difference between (**d**) and (**e**).

Figure 5 shows one example of the proposed two-step deep learning phase unwrapping method. Figure 5a–c are the input wrapped phase, output from network I, and the map of the assigned integer from network II, respectively. Figure 5d is the unwrapped phase obtained by jointly processing Figure 5a,c. For comparison, the unwrapped phase by the conventional flood-fill phase unwrapping method shown in Figure 5e is used as the reference and the difference between two unwrapped phases is plotted in Figure 5f. Only a few pixels (marked by the yellow circle in the inset of Figure 5d) have the error of 2*π*, resulting from the error from network I. This example demonstrates the effectiveness of the proposed phase unwrapping techniques.

**Figure 6:**
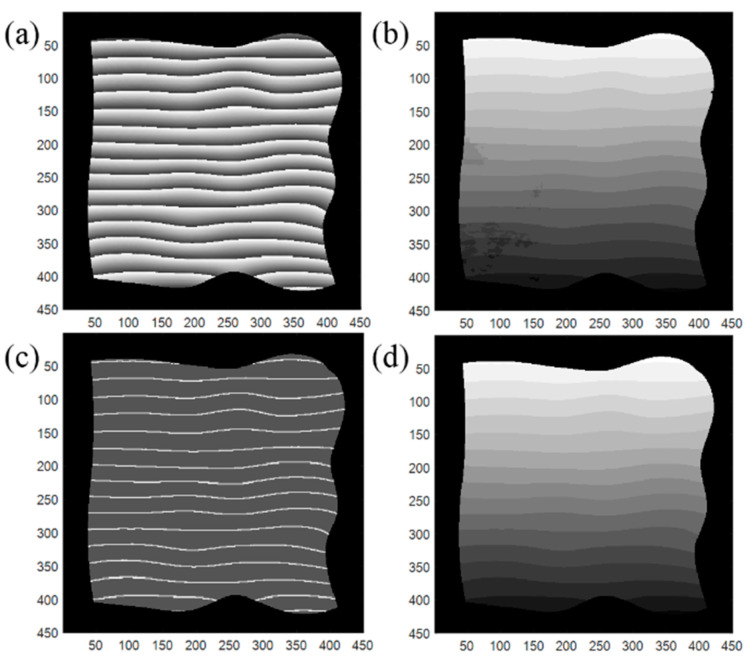
Simulation results on phase unwrapping. (**a**) Wrapped phase, (**b**) map of the assigned integer using the network architecture reported in [14,15], (**c**) segmented image with 4 labels from network I, (**d**) map of the assigned integer from network II.

Figure 6 demonstrates one advantage of the proposed method over the method of directly assigning the integer level to each segment of the wrapped phase in fringe projection 3D imaging. Figure 6a is the wrapped phase, and Figure 6b is the map of the assigned integer using the network architecture reported in [14,15]. Some errors can be found in the bottom left corner in Figure 6b. The outputs using the proposed method are shown in Figure 6c,d. Figure 6c is the segmented image with four labels from network I. From the map of the assigned integer from network II, the error is effectively suppressed. The accuracies of the unwrapped phase are 98.24% and 99.98%, respectively, using directly training and the proposed two-step training method.

**Figure 7:**
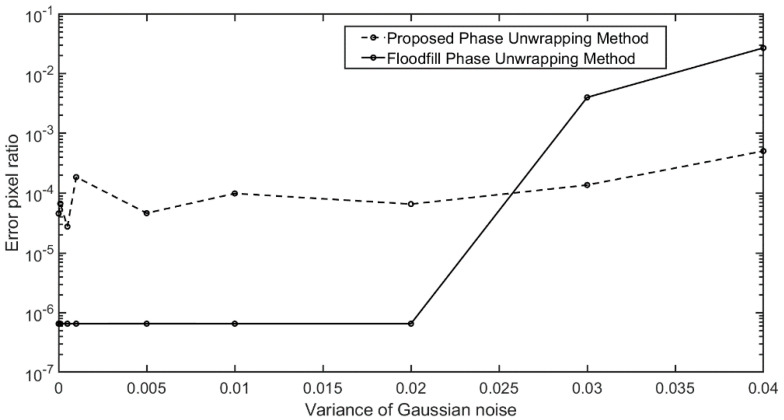
Error pixel ratio of the unwrapped phase for input dataset with different Gaussian noises.

To evaluate the robustness of the proposed method, we add the Gaussian noise into the training dataset with different variances and compare the calculated results with flood-fill phase unwrapping method. Figure 7 shows the error pixel ratios (the number of pixels with error over all pixels) of the unwrapped phases with different Gaussian-noises. When small Gaussian noise (variance smaller than 0.02) is added to the wrapped phase, the error of the flood-fill phase unwrapping method is much lower than the proposed method. By examining the error map, we find that, although the number of error pixels is more than that in flood-fill method, most of the error pixels are located on the edge of the phase map and have little impact on 3D reconstruction, which will be illustrated in experiments. When the Gaussian noise becomes severe, the error of the flood-fill method increases rapidly, while the error of the proposed method remains almost unchanged. It is illustrated that the proposed method is robust to noise.

## 3. Experiments

In this section, the effectiveness of the model trained by simulation dataset on experimental data is demonstrated. We will first evaluate the performance with a simple object, and then demonstrate that the proposed neural network can process the wrapped phase with the maximum number of phase regions much larger than that of the training data. With isolated objects, we will also validate another key feature that the size of the phase map is not limited by that of the training data.

**Figure 8:**
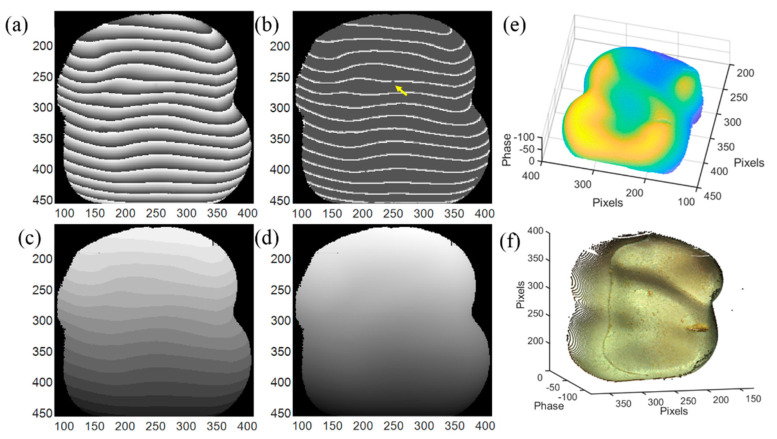
Phase unwrapping and 3D reconstruction of a tooth model. (**a**) Input wrapped phase data, (**b**) output phase feature map, (**c**) output phase feature map with assigned integer to each segment, (**d**) unwrapped phase, (**e**) 3D construction image of the target, and (**f**) 3D image of the target with color information.

**Figure 9:**
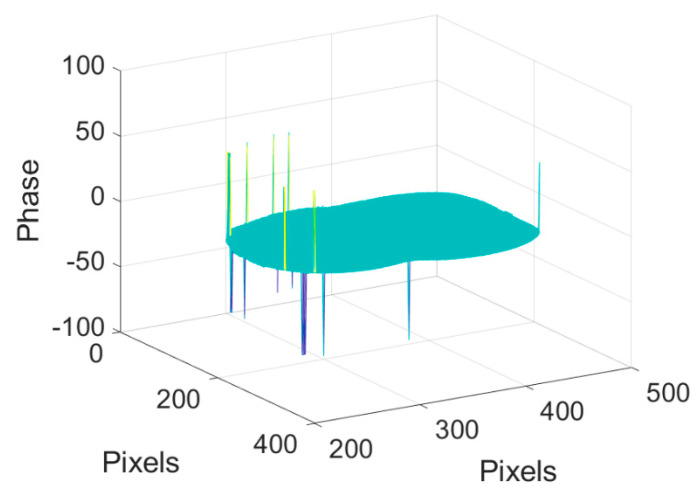
The error of the 3D reconstruction of Figure 8.

First, a tooth model is used as a measurement sample. The phase unwrapping process and 3D reconstruction result are shown in Figure 8, where (a)–(d) are the input wrapped phase, output phase feature map, output phase feature map with assigned integer to each segment, and unwrapped phase, respectively. The number of the wrapped phase segments is 15, less than the maximum number of the phase segments in the training dataset. In the output phase feature map shown in Figure 8b, some disconnected labels (located by the yellow arrow) may exist due to the experimental noise. Those disconnected labels will cause the counting error in network II; therefore, before sending the outputs to network II, a detection algorithm of the disconnected labels is added to eliminate this error by reconnecting the disconnected labels, and this can be easily realized by morphological operation carried in OpenCV module in Python. The integer map from network II is shown in Figure 8c and the unwrapped phase is shown in Figure 8d. By subtracting the unwrapped phase from the background, the phase of the tooth model is plotted in Figure 8e [20]. Figure 8f is the color 3D reconstruction image of the tooth model combined with color information. The contour and the variation of the surface are fully reconstructed, even the defect in the right region is clearly recovered. The error of the 3D reconstruction of Figure 8 compared with GT is shown in Figure 9. It can be seen that the error is mainly located on the edge of the 3D surface and have little impact on the display of the 3D reconstruction.

**Figure 10:**
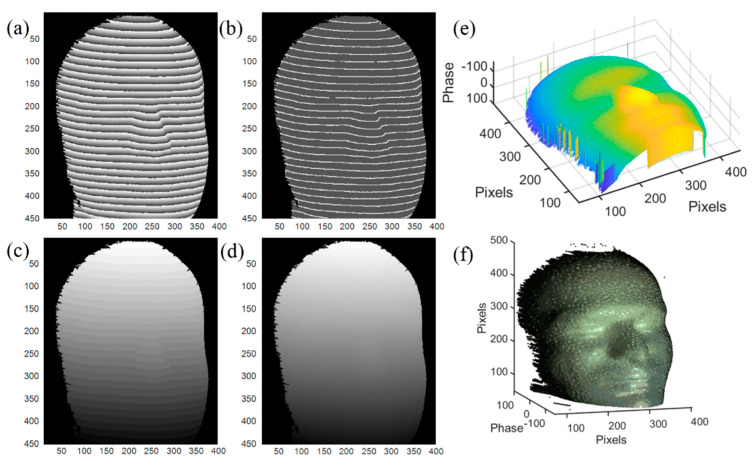
Phase unwrapping and 3D reconstruction of a human head model with thinner sinusoidal stripes. (**a**) Input wrapped phase data; (**b**) output phase feature map, (**c**) output phase feature map with assigned integer to each segment, (**d**) unwrapped phase, (**e**) 3D construction image of the target, and (**f**) 3D image of the target with color information.

To illustrate that the proposed deep learning phase unwrapping method is still robust when the wrapped phase segments is more than the maximum number of phase segments in the trained neural network, we measure a human head model made of foam as shown in Figure 10, where the number of the wrapped phase segments is 30, much larger than 17 in the training dataset. Figure 10b is the output phase feature map with few disconnected labels, and Figure 10c,d are the integer map from network II and the unwrapped phase. Similarly, the 3D reconstruction image directly based on phase map is shown in Figure 10e. 3D surface of the human head model combined with color information is reconstructed in Figure 10f. Since the sinusoidal fringe period is smaller, more detail information of the reconstructed image can be obtained.

**Figure 11:**
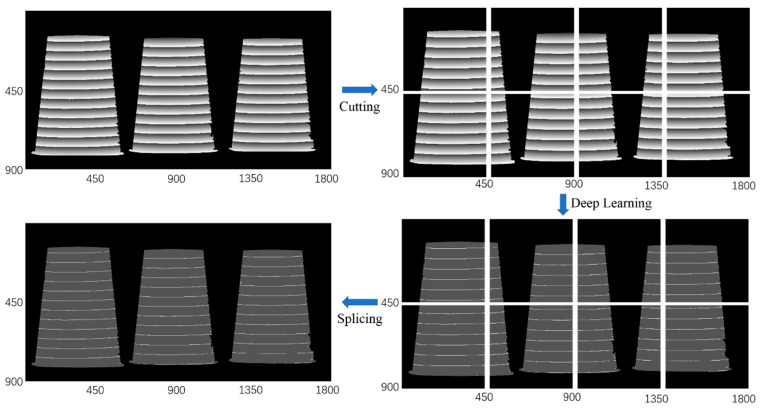
The workflow of phase unwrapping for large image size.

One common issue in the reported deep learning phase unwrapping methods is that the image size has to be the same as the size of image in the training dataset, due to the fact that these networks are not fully convolutional neural network, as shown in Figure 3. Unlike other deep learning applications, wrapped phase cannot be resized into the lower resolution because this process will blur the phase boundary of the wrapped phase. In the proposed deep learning phase unwrapping method, the output phase feature map from network I is labeled with only four parameters. Therefore, we can cut the large wrapped phase map to several segments with the same size as that of the trained data, send them to the trained neural network, and then stitch output phase feature maps together as shown in Figure 11. If the wrapped phase map cannot be exactly cut to segments with the same size of the trained data, we can simply add 0 into the small segments so that the image size is the same as the training data. We can also cut the wrapped phase map to segments with some overlaps so that each segment has the same size as the trained data. In network II, the assigned integer is fixed in the training process with the initial integer as 0. Therefore, it should be noted that, if one large image is cut into several pieces, each piece will have the same initial integer. Therefore, it is necessary to make one output image as a reference and add appropriate bias parameter to other output images according to the relationship between them and the reference image.

**Figure 12:**
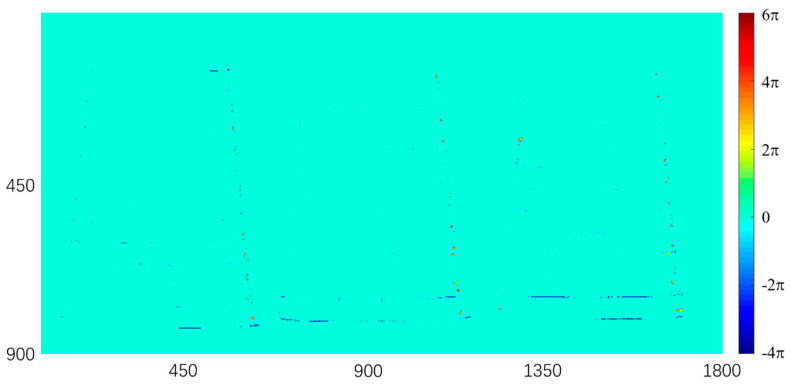
The segmentation error between the output feature map and the ground truth.

To demonstrate this key feature, we image three paper cups placed at different depths. Figure 11 shows the workflow of the phase unwrapping, the spatial resolution of the wrapped phase is 900 × 1800, much larger than the dimension (450 × 450) of the trained images. The large wrapped phase is first cut to 2 × 4 segments with the dimension of 450 × 450 pixels, and then sent to the trained network I. The outputs from network I can be rebuilt into one image (900 × 1800) by a splicing process, as shown in Figure 11. Compared to the GT, the segmentation error of the final phase feature map reconstructed from the whole surface is plotted in Figure 12. The ratio of the error pixels from the whole pixels is 8.45 × 10^𢈒4^ and most error pixels are located on the boundary of adjacent segmented phase regions, demonstrating the effectiveness of the proposed deep learning phase unwrapping method in processing the large-size image. The trained neural network can automatically recognize the discontinued wrapped phase regions and correctly segment the wrapped phase with four features. This method is very convenient in fringe projection 3D imaging with discrete objects.

**Figure 13:**
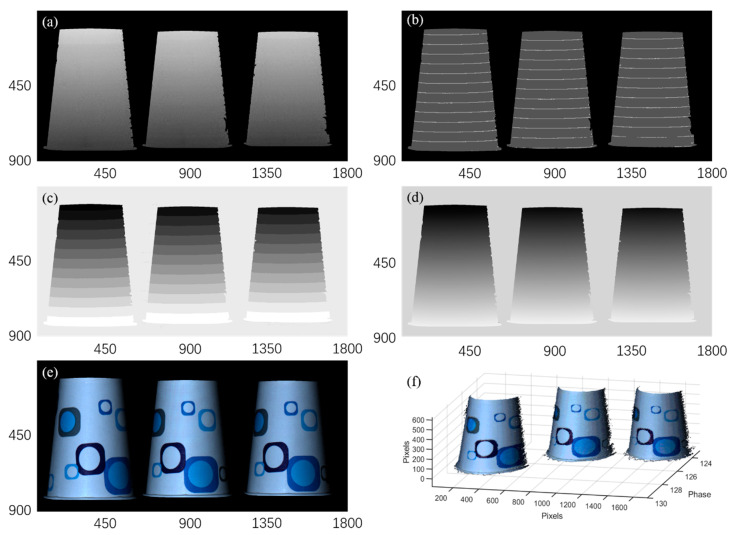
3D reconstruction of the isolated objects using the proposed neural network. (**a**) Phase distribution of one single period; (**b**) the output label image from network I; (**c**) the integer calculated using the output label image with the help of (**a**); (**d**) the unwrapped phase distribution; (**e**) the 2D color image for comparison; and (**f**) 3D reconstruction using (**c**,**d**).

It should be noted that the isolated wrapped phases cannot be correctly unwrapped independently. Typically, one needs to obtain the relative position between isolated objects first with the low frequency of the projected fringes. Figure 13a is the phase distribution of the three paper cups with only one phase region. With the help of Figure 13a, we can transform the phase feature map obtained from the phase shift fringe with high frequency (as shown in Figure 13b) to the phase feature map with appropriately assigned integer to each segment (as shown in Figure 13c). Using Equation (1), the unwrapped phase can be finally obtained, as shown in Figure 13d. Based on Figure 13d, one can reconstruct the 3D surfaces of the three paper cups by subtracting the phase of the background [20], and clearly see the depth of each paper cup. Note that, here we only show the relative position of the three paper cups. If one needs to obtain the absolute distance between each paper cup, the calibration should be done first [21]. Figure 13e is the 2D color image of the paper cups for comparison and Figure 13f is the reconstructed 3D surface.

## 4. Conclusions

We have proposed a new deep learning phase unwrapping method for fringe projection 3D imaging. Instead of labeling the phase segment with integers directly, we label the transition edges of the wrapped phase. The key advantages are (1) The use of four specific features ensures the accurate segmentation of the wrapped phase with serious Gaussian noise; (2) the image size is unlimited. Normally, these two advantages are especially suitable for fringe projection 3D imaging applications. As current, two steps are needed to unwrap the phase with disconnected object. We will develop new neural networks to address this issue in the near future.
